# Changes in Medical Education in 21 ^st^ Century

**DOI:** 10.1055/s-0045-1802318

**Published:** 2025-01-28

**Authors:** Harish Gupta

**Affiliations:** 1 Department of Medicine, King George's Medical University, Lucknow, Uttar Pradesh, India

*“Ensure inclusive and equitable quality education and promote lifelong learning opportunities for all.* ”

–United Nation's Sustainable Development Goal Number 4. 1

Dear Editor,

Bhattacharya et al assert that as (medical) students have changed (in this era), there is a case for medical teachers to change too in their article published on November 20, 2024. 2 The authors go through electronic modes of new age learning, available digital technologies, apps in vogue, and software run on various platforms to make their case. The authors highlight that youngsters of this era are comfortable with these devices and we should bridge the digital divide for constructing an equitable and fair playing field. They further underscore that various tools and Web sites provide new avenues of learning and we should explore their potential to enhance learning opportunities for the budding medicos and residents. I agree with all the points and arguments in favor of the proposition. When the digital era is overlapping and we are about to be immersed in artificial intelligence and virtual reality, we need more perspective for medical education to help our workforce-in-training so as to widen their horizons of knowledge.

Nonetheless, there are few points of disagreement in the Editorial where I put on the table a different view with its reasoning. Under a header of *How can teachers adapt* , the writer states that contents available on the web are not authentic, hence teachers should guide the learners in the knowledge acquisition. Nevertheless, another sort of divide has appeared on the web due to this reason—paid content. After vetting the information and input nowadays, editors and hosts create a space akin to a walled garden. One can access the contents on a price. 3 Many a publishers justify the model by citing various reasons, 4 while defenders of open-access liberty oppose the strategy, which puts the global south at a position of disadvantage. 5

Some publishers have come up with e (only) contents. 6 In a textbook, some chapters are printed on paper, while others are available (only) in a soft copy. An interested reader has to access these electronic chapters only through his or her device. For example, our Bible— *The Harrison's Principles of Internal Medicine* —has part 21 of video collection and part 24 of clinical procedure tutorials. 7 One cannot reproduce these chapters in any other format, and hence are unique. Although access to certain contents demands a password/clinical key/passcode/digital account, others are available in the public domain. Such creativity has its own limitations and drawbacks beside what the authors state in the article. This new age division between haves and have nots may have long-term implications on the basis of purchasing power and access to resources, which need immediate attention. We should put our heads together given the enormity of the challenge and should not avoid the elephant in the room.

Certain educational institutions and universities made contracts with publishers to allow institutional access to their contents. 8 Their open-access journal and publication are open to the subscribing institution *only* . Some journals allow access to patients' data of their trial to all after a certain duration/conditions. 9 It appears that entrepreneurs and capitalists, due to their commercial interests, want to earn dividends by limiting access to those who can pay, while crusaders of open access pull the tug of war toward another direction. History will remember this epoch with much interest whatever its results are.

And history will remember who stood where, for what, and with whom!
